# Age-Associated Changes in Monocyte and Innate Immune Activation Markers Occur More Rapidly in HIV Infected Women

**DOI:** 10.1371/journal.pone.0055279

**Published:** 2013-01-24

**Authors:** Genevieve E. Martin, Maelenn Gouillou, Anna C. Hearps, Thomas A. Angelovich, Allen C. Cheng, Fiona Lynch, Wan-Jung Cheng, Geza Paukovics, Clovis S. Palmer, Richard M. Novak, Anthony Jaworowski, Alan L. Landay, Suzanne M. Crowe

**Affiliations:** 1 Centre for Virology, Burnet Institute, Melbourne, Australia; 2 Department of Medicine, Monash University, Melbourne, Australia; 3 Centre for Population Health, Burnet Institute, Melbourne, Australia; 4 School of Applied Sciences, RMIT University, Melbourne, Australia; 5 Infectious Diseases Unit, Alfred Hospital, Melbourne, Australia; 6 Department of Medicine, University of Illinois at Chicago, Chicago, Illinois, United States of America; 7 School of Medical Sciences, University of New South Wales, Sydney, Australia; 8 Department of Immunology, Monash University, Melbourne, Australia; 9 Department of Immunology/Microbiology, Rush University Medical Center, Chicago, Illinois, United States of America; Karolinska Institutet, Sweden

## Abstract

**Background:**

Aging is associated with immune dysfunction and the related development of conditions with an inflammatory pathogenesis. Some of these immune changes are also observed in HIV infection, but the interaction between immune changes with aging and HIV infection are unknown. Whilst sex differences in innate immunity are recognized, little research into innate immune aging has been performed on women.

**Methods:**

This cross-sectional study of HIV positive and negative women used whole blood flow cytometric analysis to characterize monocyte and CD8^+^ T cell subsets. Plasma markers of innate immune activation were measured using standard ELISA-based assays.

**Results:**

HIV positive women exhibited elevated plasma levels of the innate immune activation markers CXCL10 (p<0.001), soluble CD163 (sCD163, p = 0.001), sCD14 (p = 0.022), neopterin (p = 0.029) and an increased proportion of CD16^+^ monocytes (p = 0.009) compared to uninfected controls. Levels of the innate immune aging biomarkers sCD163 and the proportion of CD16^+^ monocytes were equivalent to those observed in HIV negative women aged 14.5 and 10.6 years older, respectively. CXCL10 increased with age at an accelerated rate in HIV positive women (p = 0.002) suggesting a synergistic effect between HIV and aging on innate immune activation. Multivariable modeling indicated that age-related increases in innate immune biomarkers CXCL10 and sCD163 are independent of senescent changes in CD8^+^ T lymphocytes.

**Conclusions:**

Quantifying the impact of HIV on immune aging reveals that HIV infection in women confers the equivalent of a 10–14 year increase in the levels of innate immune aging markers. These changes may contribute to the increased risk of inflammatory age-related diseases in HIV positive women.

## Introduction

HIV positive individuals are at a greater risk than their seronegative counterparts of a range of conditions usually associated with aging such as cardiovascular disease [1], osteoporosis [2], frailty [3] and neurocognitive decline [4]. These conditions are associated in the elderly with elevated levels of inflammatory markers including IL-6 and TNF [5], [6]. Thus the finding that young HIV positive and elderly individuals show similar elevations of these markers [7], [8] suggests that systemic chronic inflammation may be a common feature of both HIV infection and aging.

Studies of HIV-related immune senescence have mainly focused on the adaptive immune system and identified markers of adaptive immune aging including expansion of CD28^−^CD57^+^ CD8^+^ T cells and shortened telomeres in CD8^+^ T cells [9], [10] occur prematurely in younger HIV positive individuals [11], [12]. However, expansion of a subset of monocytes that express CD16 and that are reported to have an activated and inflammatory phenotype [13] has been demonstrated in both aged [14], [15], [16] and HIV positive individuals [17], [18]. This suggests that immune dysfunction in both aging and HIV extends also to innate immunity.

We and others have shown that soluble plasma markers of innate immune activation including soluble CD163 (sCD163) [17], [19], soluble CD14 (sCD14) [20], CXCL10 (also known as interferon inducible protein 10 or IP-10) [8], [17], [21] and neopterin [17], [22] are elevated in HIV infection. However, these findings were observed in cohorts consisting either predominantly or exclusively of men, with only one study focusing on females [8]. In HIV positive individuals, plasma CXCL10 correlates with the proportion of CD16^+^ monocytes [21], which suggests a critical link between monocytes and HIV related innate immune activation and dysfunction.

Sex-associated differences in monocyte phenotype and plasma markers indicate that innate immune changes should be considered separately in males and females. Females have a lower proportion of non-classical (CD14^+^CD16^++^) monocytes than males [14], [23] and monocytes from the two sexes have different expression patterns of the surface markers CD38, CD62L and CD115 on monocyte subsets [14]. We have recently shown that, when adjusted for age, healthy females have elevated plasma levels of CXCL10 and sCD163 and decreased sCD14 compared with males [14]. Adaptive immune activation in HIV infection also differs significantly between the sexes. *In vitro*, T cells from healthy men and women produced different levels of interferon-γ (IFNγ) in response to stimulation with anti-CD3 and anti-CD28 antibodies [24]. Adaptive immune activation in HIV infection differs significantly between the sexes. Females have significantly higher percentages of activated CD8^+^ T cells (CD38^+^/HLA-DR^+^) than their male counterparts when matched for viral load [25], highlighting the need to consider sex as a variable in immunological HIV studies. These findings indicate sex is a significant variable in immunological responses, however sex differences in monocyte function have not been investigated in the context of HIV infection to date.

In this study, we sought to investigate the impact of both age and HIV infection on biomarkers of innate immune activation that are relevant to age-associated diseases in women and determine whether changes in these markers are independent of those of the adaptive immune system.

## Methods

### Participant recruitment

This study was approved by The Alfred Hospital Ethics Committee, the Monash University Human Research Ethics Committee, Rush University Medical Center (RUMC) Institutional Review Board and the University of Illinois at Chicago (UIC) Institutional Review Board.

HIV positive women aged between 20 and 63 years (n = 23) were recruited through the UIC Medical Centre and RUMC infectious diseases clinics (Chicago, IL, United States). A control group of healthy, HIV negative women aged between 20 and 82 years (n = 53) was recruited from the community (Melbourne, Australia n = 30 and Chicago, IL, United States n = 23). Women who were pregnant, using anti-inflammatory drugs (including steroids and non-steroidal anti-inflammatory drugs) on a daily basis, had active malignancy, current infection or history of trauma or vaccination in the three weeks prior to study date were excluded from participation.

Written, informed consent was obtained from participants. Participation involved collection of a single blood sample and completion of a questionnaire requesting demographic, health and lifestyle information, including a validated menopausal staging algorithm [26].

### Monocyte and lymphocyte phenotyping

Whole blood phenotyping was performed on EDTA-anticoagulated blood within 2 hours of blood collection by a single individual who worked in both the Australian and the US laboratories. Standard operating procedures for every assay were used in the two laboratories to ensure comparability of analyses. Erythrocytes were lysed using 20x volume of BD FACS Lysing Solution and subsequently washed twice (450x *g* for 5 minutes at 4°C) with FACS wash (calcium and magnesium free phosphate buffered saline (PBS-) pH 7.4, 2 mM EDTA, supplemented with 1% heat inactivated fetal or newborn calf serum (FCS/CCS) or 0.5% bovine serum albumin (BSA)).

Cells were incubated in the dark on ice for 30 minutes with pre-titrated volumes of monoclonal antibodies specific for CD14 (clone M5E2, APC, BD Pharmingen), CD16 (3G8, PE-Cy7, BD Pharmingen), CD3 (SK7, PerCP or PerCP-Cy5.5, BD), CD8 (SK1, PE or APC, BD), CD28 (CD28.2, APC or PE, BD Pharmingen) and CD57 (NK-1, FITC, BD). Following staining, cells were washed once with FACS wash (450x *g* for 5 minutes at 4°C), fixed with 1% formaldehyde and stored in the dark at 4°C before analysis by flow cytometry.

Data were acquired on a FACSCalibur (BD Biosciences) in both laboratories. Post-acquisition compensation was performed using singly-stained cell samples; data were analyzed by the same person for all samples using Gatelogic (Inivai Technologies) software. Monocytes and lymphocytes were initially defined using an appropriate forward scatter (FSC) and side scatter (SSC) gate. CD14 and CD16 were used to gate monocytes into CD14^++^CD16^−^ (classical) and CD14^var^CD16^+^ (CD16^+^, non-classical and intermediate) populations. CD3 and CD8 were used to define CD8^+^ T cells and expression of CD57 and CD28 was assessed relative to isotype controls. Values derived from plots with fewer than 200 events were excluded from statistical analyses.

Analyses were performed using the proportion of CD16^+^ monocytes, as well as using data that further divides this subset into non-classical (CD14^+^CD16^++^) and intermediate (CD14^++^CD16^+^). However, as similar patterns were seen for both non-classical and intermediate subsets, only results for CD16^+^ monocytes are presented here.

### Measurement of soluble plasma markers

Plasma was separated from EDTA-anticoagulated blood and stored at −80°C or −140°C. Plasma samples were thawed once and clarified by centrifugation (10,000x *g* for 10 minutes) prior to measurement of soluble plasma markers. Commercial ELISA kits used as per manufacturer's instruction were utilized for the measurement of sCD163 (IQ products, Cat. #IQP-383), neopterin (Screening EIA, Brahms, Cat. #99R.096), sCD14 (Quantikine, R&D Systems, Cat. #DC140) and CXCL10 (Quantikine, R&D Systems, Cat. #DIP100). Plasma samples were diluted 1∶10 and heat inactivated (80°C for 10 minutes) prior to measurement of lipopolysaccharide (LPS) levels using the chromogenic Limulus Amebocyte Lysate kit (Lonza, Cat #50–647U). Measurement of each marker was conducted on all samples at the same time, by the same person on batched frozen samples.

### Statistical analyses

Comparisons between groups were made using the Students *t* test or the Mann-Whitney *U* test as appropriate. Linear regression models adjusted on HIV status for each parameter were fitted with age as an outcome. Age was used as an outcome to allow for identification of parameters independently associated with age using multivariable modeling. In order to differentiate from the multivariable analyses which includes several parameters in the same model, these models are referred to as “bivariable analyses” hereafter. To assess whether the parameter levels compared to age differed by HIV status, an interaction model whereby each group had its own slope for the parameter value over age (stratified by HIV status) was compared to a model of common slope (non-stratified model) using the likelihood ratio test. The parameters significant in the bivariable analyses were entered in a multivariable linear regression model and through a process of stepwise elimination only significant, independent correlates of age were retained in the final model. Analyses were performed using Stata Version 11. *p* values <0.05 were considered significant.

## Results

### Participants

Characteristics of the 23 HIV positive women and the 53 HIV negative women are shown in Table 1. Women with HIV infection had a higher body-mass index. There was no significant difference in history of recreational drug use (*p* = 0.724) or menopausal status (*p* = 0.21) between the two groups.

**Table 1:** Comparison of demographic variables between HIV positive and HIV negative groups.

	HIV negative	HIV positive	*p* value
N	53	23	-
Median age (range) years	47 (20–82)	40 (20–63)	0.069
Median BMI (range)	23.8 (18.0–42.0)	30.6 (19.4–43.9)	**<0.001**
Viral load (copies/mL)			
– Detectable viral load, >50 copies/mL (n (%))	-	8 (34.8)	-
– Median viral load (range) a	-	2256 (208–1.28×10^6^)	-
CD4 count (cells/µL)			
– Median CD4^+^ T cell count (range)	-	433 (4–1433)	-
– Median nadir CD4^+^ T cell count (range)	-	123 (1–688)	-
Hepatitis C virus (HCV) positive (n(%)) b	-	6 (27.3)	-
On cART (n (%))	-	20 (87.0)	-

a median and range shown of those patients with detectable viral load.

b HCV status unknown for 1 patient.

Abbreviations: SD, standard deviation; BMI, body mass index; cART, combination antiretroviral therapy.

Continuous variables were compared using the Students *t* test or the Mann-Whitney *U* test as appropriate. *p* values<0.05 were considered significant.

### HIV infection affects age-related changes to innate immune activation markers

The effect of HIV infection on levels of innate immune activation markers was determined using linear regression analysis. Plasma levels of sCD163 (*p* = 0.001), sCD14 (*p* = 0.022), neopterin (*p* = 0.029) and CXCL10 (*p*<0.001) were significantly elevated in HIV positive compared with HIV negative women (Table 2). The proportion of CD16^+^ monocytes was also higher in HIV positive women (*p* = 0.009). No difference was observed in plasma LPS levels.

**Table 2:** Linear regression analyses to examine the relationship of each parameter with age and the impact of HIV infection on this relationship.

		*Parameter*			*HIV status*		
Parameter	n	Coefficient	95% CI	*p* value (parameter) a	Coefficient	95% CI	*p* value (HIV status) b
Monocyte subsets							
- % of monocytes that are CD16^+^	76	0.59	0.12, 1.06	**0.015**	−10.55	−18.38, −2.73	**0.009**
Plasma markers							
- sCD163 c	75	0.01	0.01, 0.02	**0.002**	−14.47	−22.71, −6.23	**0.001**
- sCD14	65	0.00	−0.00, 0.01	0.373	−11.13	−20.58, −1.68	**0.022**
- LPS	71	−4.66	−18.63, 9.31	0.508	−6.60	−14.66, 1.46	0.107
- CXCL10 d	76	0.17	0.09, 0.25	**<0.001**	2.20	−11.23, 15.64	0.745
- Difference in slopes e		−0.15	−0.24, −0.06	**0.002**	-	-	**-**
- HIV positive	23	0.02	−0.00, 0.05	0.056	-	-	**-**
- HIV negative	53	0.17	0.08, 0.26	**<0.001**	-	-	**-**
- Neopterin	73	1.05	−1.27, 3.38	0.369	−9.60	−18.21, −0.99	**0.029**
Lymphocyte subset							
- CD28^−^CD57^+^	75	0.26	0.02, 0.50	**0.034**	−12.62	−21.57, −3.66	**0.006**

Bivariable regression analyses shown are performed on combined HIV negative and HIV positive participants with age as an outcome, adjusting for HIV status. An interaction model whereby each group had its own slope for the parameter value over age (stratified by HIV status) was compared to a model of common slope (non-stratified model) using the likelihood ratio test and the model of best fit is shown.

a *p* value that the slope of the line is non-zero.

b *p* value that the intercept of the line is different for HIV positive and HIV negative.

c One outlier was excluded from this analysis.

d The model with the best fit for CXCL10 was stratified by HIV status and as such the slopes for HIV negative and HIV positive groups are different and are shown separately.

e This term describes the difference in slope between the HIV positive and HIV negative models. The *p* value indicates that the slopes are significantly different.

Note. *p* values <0.05 were considered statistically significant.

Abbreviations: CI, confidence interval; LPS, lipopolysaccharide.

**Figure 1:**
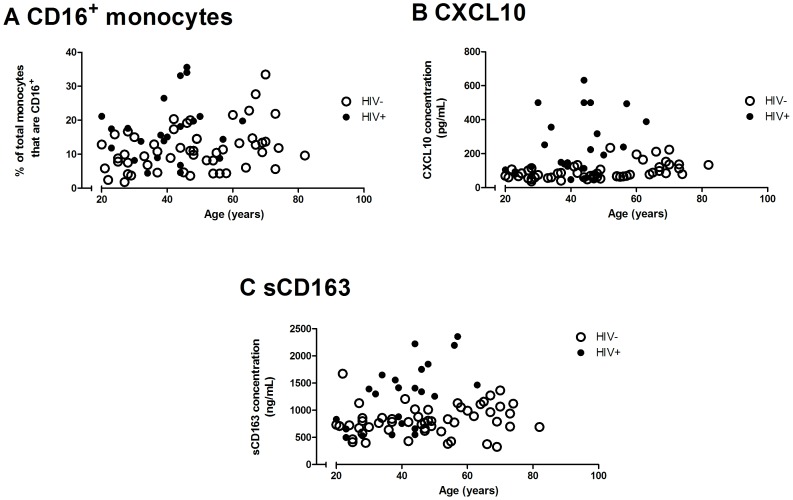
Bivariable linear regression of plasma CXCL10 and the proportion of CD16^+^ monocytes. A comparison of the slopes between HIV negative (open circles, dashed line; n = 53) and HIV positive (closed circles, solid line; n = 23) groups and the relationship with age for (a) % CD16^+^ monocytes determined by whole-blood flow cytometry, (b) plasma CXCL10 and (c) sCD163 levels determined by ELISA.

Linear regression analyses were also performed to assess the relationship between each innate immune parameter and age. The stratified model was preferred for CXCL10 (*p* = 0.002, see Table 2 and Figure 1) while for all the other parameters, the non-stratified model was shown to have a better fit. The slope of age-related changes in CXCL10 is altered in HIV positive compared to HIV negative women (Table 2). Soluble CD163 and the proportion of CD16^+^ monocytes were shown to increase significantly with age (*p* = 0.002 and *p* = 0.015 respectively, Table 2 and Figure 1). Age-related increases in the proportion of CD57^+^ (*p* = 0.005) and CD28^−^ (*p* = 0.025) CD8^+^ T cells were demonstrated (data not shown) and were reflected in changes in the CD28^−^CD57^+^ CD8^+^ T cell subset (*p* = 0.034, Table 2), a well-characterised biomarker of adaptive immune senescence.

The coefficient of the HIV term (shown in Table 2 under “HIV status”) gives a quantitative measure of the average age difference between the two groups for those parameters fitted with a non-stratified model. Although the slope (i.e. rate) of increase with age is the same, the proportion of CD16^+^ monocytes in HIV positive women of any given age is similar to those of seronegative women 10.6 years older (*p* = 0.009, Table 2). In the same way, HIV positive women have plasma levels of sCD163 similar to those of seronegative women 14.5 years older (*p* = 0.001). HIV positive women have similar levels of CD28^−^CD57^+^CD8^+^ T cells to seronegative women aged 12.6 years older (Table 2).

### Increases in plasma CXCL10 and sCD163 are independent of changes in CD8^+^ T cells

We next determined whether the observed HIV-related innate immune changes were independent of adaptive immune changes and thus whether their inclusion into immunogerontological study designs would provide additional information beyond that indicated by T cell markers alone. Multivariable linear regression modeling was performed on variables identified as significant in bivariable analyses as associated with age when adjusted for HIV status. The model with best fit analyzing only innate parameters contained both sCD163 and CXCL10 (n = 75, Akaike information criterion = 602.53, *R^2^_adj_* = 0.30; data not shown) demonstrating that plasma sCD163 and CXCL10 concentrations independently increase with age.

The optimum model constructed using all parameters (both innate and adaptive) is shown in Table 3. The inclusion of CXCL10 and sCD163 alongside total CD28^−^ and CD28^+^CD57^−^ CD8^+^ T cells indicates that the observed age-related increases in CXCL10 and sCD163 are independent of changes in CD8^+^ T cell subsets.

**Table 3:** Multivariable linear regression model.

Parameter	Coefficient	95% CI	p value
CXCL10 a	0.18	0.10, 0.25	**<0.001**
– Difference in slopes b	−0.18	−0.26, −0.10	**<0.001**
– HIV positive	0.00	−0.02, 0.03	0.771
– HIV negative	0.18	0.08, 0.25	**<0.001**
sCD163	0.01	0.00, 0.02	**0.033**
CD28^+^CD57^−^ CD8+ T cells	−1.12	−1.74, −0.49	**0.001**
Total CD28^−^ CD8+ T cells	−1.01	−1.65, −0.36	**0.003**
HIV status	0.04	−12.36, 12.45	0.995
Constant	122.33	64.60, 180.28	**<0.001**

Combinations of parameters identified as significant in bivariable modeling were included in multivariable linear regression models, with stepwise elimination of variables that were not statistically significant in the model. n = 74, Akaike information criterion (AIC) = 585.15, *R^2^_adj_* = 0.40.

a The model with the best fit for CXCL10 was stratified by HIV status and as such the slopes for HIV positive and HIV negative groups are different and are shown separately.

b This term describes the difference in slope between the HIV positive and HIV negative models. The *p* value indicates that the slopes are significantly different.

Note. *p* values <0.05 were considered statistically significant.

Abbreviations: CI, confidence interval; LPS, lipopolysaccharide.

## Discussion

Here we show that several markers of innate immune activation and aging occur prematurely in HIV positive women. Of these, the increased proportion of CD16^+^ monocytes and plasma sCD163 appear approximately 10–14 years earlier in HIV positive compared to HIV negative women. These novel findings demonstrate an increase in the rate of age-related changes (as shown here with CXCL10) in women during HIV infection.

We have recently shown that levels of innate immune activation markers including sCD163 and CXCL10 in young, HIV positive males are similar to those in elderly seronegative men [17]. Whilst many studies demonstrate similarities between HIV and age-related immune changes, few studies address the interaction between these two factors. To do this, we analysed data with age as a continuous variable and compared the trajectory in innate immune changes between HIV positive and negative women. Our finding of an age-related increase in sCD163 levels (a marker of monocyte activation) and inflammatory CD16^+^ monocytes in both HIV positive and HIV negative women is consistent with previous findings in men/mixed cohorts [14], [15], [16], [17]. We and others have previously shown HIV-related increases in both sCD163 levels and CD16^+^ monocytes [17], [18], [19]. In the present study we quantified this difference and found HIV positive women have levels of sCD163 and CD16^+^ monocytes equivalent to those in seronegative women aged on average 14.47 or 10.55 years older, respectively. Such premature changes in the inflammatory milieu may contribute to the early development of age-related diseases in HIV positive individuals.

In contrast, age-related increases in CXCL10 concentrations were found to occur at an accelerated rate in HIV positive women, suggesting age and HIV may act synergistically to increase CXCL10 levels. CXCL10 is an inflammatory chemokine which has been associated with risk of coronary heart disease [27] and myocardial infarct size [28] in the general population. Additionally, a direct role for CXCL10 in the development of atherosclerotic plaques in mice has been demonstrated [29]. Thus, the finding that HIV acts synergistically with age to increase CXCL10 levels may have implications for the development of cardiovascular disease in HIV positive individuals.

Multivariable modeling indicated that age-related changes to sCD163 and CXCL10 were independent, and furthermore that these innate immune changes were independent of previously demonstrated changes in the proportion of CD28^+^CD57^−^ CD8^+^ T cells indicative of adaptive immune senescence. This novel finding demonstrates that in aging and HIV infection, adaptive and innate changes are independent of one another. This finding suggests that parallel but independent mechanism may be driving innate and adaptive immune changes during HIV infection and justifies the inclusion of innate immune parameters, including CXCL10 and sCD163, alongside adaptive immune parameters in studies of immune senescence.

In addition to increases in sCD163 and CXCL10 levels, we also found elevated plasma levels of neopterin (a GTP metabolite produced by macrophages following IFN-γ stimulation [30]), in HIV positive women, similar to our previous observation in males [17]. Unlike our findings in males, we found significantly elevated levels of sCD14 in HIV positive women which has been linked to HIV-related mortality [31]. Increased sCD14 levels have been previously shown in some [20], [32] but not all [19] studies in HIV positive adults and in a pediatric HIV cohort [33]. sCD14 is required for LPS stimulation of TLR4 and is shed from the surface of monocytes following activation [34]. In HIV positive individuals, sCD14 has been associated with elevated LPS levels related to microbial translocation across the gut mucosa [20]. However, LPS levels within the HIV positive group were not elevated in this study. Plasma levels of LPS and sCD14 have been shown to be discrepant in other studies, suggesting the relationship between LPS and sCD14 may be complex and context-dependent [35], [36] or that LPS may be a less robust biomarker. Taken together, these results suggest that whilst HIV may have a similar effect in males and females with respect to augmenting plasma levels of CXCL10, neopterin and sCD163, HIV positive females may not exhibit significant changes in factors such as LPS. This, combined with previously reported significant differences between males and females in CD4 T cell counts at AIDS onset [37], indicate sex-related differences exist in certain aspects of HIV-related immune dysfunction. It remains to be determined whether these sex-related differences translate to differences in morbidity risk.

Drivers of immune aging remain to be fully defined. We have previously shown that although cART is associated with an improvement in innate immune dysfunction, age-related changes to innate immune markers persist despite virological suppression [17], suggesting the involvement of other factors in addition to HIV viremia. Microbial translocation and resultant endotoxemia, which persist despite viral suppression, have been postulated to contribute to persistent immune activation and senescence, however numerous other factors including reactivation of latent viruses may also be involved (see [38], [39] for review).

There are several limitations to this study. Given the relatively small number of participants, there was insufficient power to perform separate analyses for virologically suppressed and viremic individuals or to control for unmatched demographic variables between the two groups. Levels of the innate immune parameters measured here are known to be elevated in viremic as compared to virologically suppressed HIV positive males and the relationship between viral load, CD4 T cell count and innate immune activation markers in HIV positive women requires investigation in larger cohorts studies. Significant differences existed between the HIV positive and HIV negative groups with regards to BMI and co-infection with hepatitis C virus (HCV; seen in 27.3% of HIV positive participants). CXCL10 levels [8] and the level of CD8^+^ T cell activation [40] in HIV/HCV co-infected patients have been shown to be higher than in patients individually infected with either virus. Although menopausal status was not significantly different between our HIV positive and HIV negative cohorts, we cannot exclude the possibility that hormonal and menopausal variations may influence the parameters measured here. Significantly larger cohort studies would be required to investigate these more complex associations.

We have shown an increase in the rate of age-related change in CXCL10 with HIV infection. As the increases in CXCL10 and sCD163 in HIV positive women were independent of well-characterized HIV-associated CD8^+^ T cell senescence, this study highlights the importance of including innate immune markers in future investigations. The premature development of innate immune changes in the setting of HIV infection may underlie the acceleration and/or heightened risk of diseases associated with aging in this patient population.
